# A special acute care surgery model for dealing with dilemmas involved in emergency department in China

**DOI:** 10.1038/s41598-021-81347-9

**Published:** 2021-01-18

**Authors:** Dequan Xu, Yue Yin, Limin Hou, Haoxin Zhou

**Affiliations:** 1 Department of Emergency Surgery, The First Affiliated Hospital of Harbin Medical University, 23 Youzheng Street, Nangang District, Harbin, 150001 Heilongjiang People’s Republic of China; 2 Department of Thyroid Surgery, The First Affiliated Hospital of Harbin Medical University, 23 Youzheng Street, Nangang District, Harbin, 150001 Heilongjiang People’s Republic of China

**Keywords:** Diseases, Medical research

## Abstract

There was a fast growth in the number and the formation of emergency department (ED) visits in China during the twenty-first century. As a result, engaging special medical model will be essential to decompressing the ED visits. To do this, it will be important to understand which specific aspects to focus interventions on for the greatest impact. To characterize the emergency surgery patients who were seen and discharged from ED. Retrospective cohort study of hospitalized emergency surgery patients currently under the care from specialists presenting to an urban, university affiliated hospital between 01 January 2018 and 1 January 2019. This study will highlight some of the controversies and challenges and key lessons learned. During the study period there were 231,229 ED visits; 4100 of these patients were admitted for Acute care surgery (ACS) service. Multivariate analysis identified age ≧ 65 (p = 0.023; odds ratio, OR = 2.66), ACS model (p = 0.000, OR = 0.18), ICU stay (p = 0.000, OR = 118.73) as factors associated with in-hospital mortality. There was a increase in length of stay between young and elderly postoperative patients when stratifying patients by age (11.67 ± 9.48 vs 13.95 ± 9.11 p < 0.05). ED overcrowding is not just an ED problem. ED overcrowding is a systems problem requiring a systematic facility-wide multidisciplinary response. Continuous and high-quality surveillance data across China are needed to estimate the acute care surgery model which used to deal with ED overcrowding.

## Introduction

Although emergency general surgery (EGS) and trauma patients have always been an integral part of surgical care, the field of acute care surgery (ACS) as a surgical specialty is a relatively new concept. ACS was initially proposed in 2005 as an innovative model of care to facilitate timely and high-quality EGS care along with trauma and surgical critical care^1^. Prior studies have demonstrated the ACS model can improve work flow and provide more effective delivery of care for patients with common urgent general surgery diagnoses without an increased rate of complications^2^.

**Figure 1:**
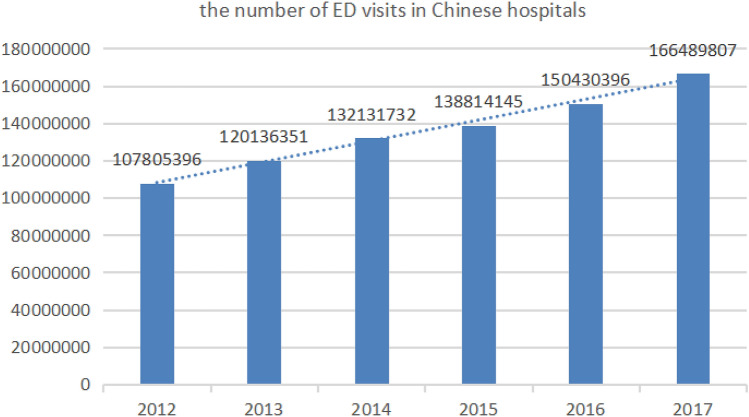
The number of ED visits in Chinese hospitals during the years 2012–2017.

After much work over the past several years, the ACS model has entered the high-speed development period. However, there is a significant burden and unmet need for acute surgical conditions in low-income and middle-income countries (LMICs), especially country like China with large population base. According to Disease Control Priorities 3 released by the World Bank, 5 billion people in the world do not have access to safe surgical and anesthesia care, with a disproportionate 9 of 10 people in LMICs^3^. Noncommunicable diseases including trauma and emergency surgery accounts for up to nearly one-third of the entire global burden of disease. In China, hospitals have noted an increased acuity of patients presenting to them. According to the “China Health Statistical Yearbook Compiled by the National Health and Family Planning Commission”, the number of emergency department (ED) visits in Chinese hospitals has increased significantly from 107,805,396 in 2012 to 166,489,807 in 2017 over a 6-year period (http://www.yearbookchina.com/) (Fig. 1). Due to high volumes of emergency surgery, a special ACS model was developed at a Third Grade Class A hospital in Harbin, Heilongjiang province, China. The aim of this study was to describe the structure of the ACS service in our hospital, the epidemiology of ACS and summarize experience to improve the efficiency of ACS service**.**

## Materials and methods

A retrospective study was conducted on patients who visited the ED at the First Affiliated Hospital of Harbin Medical University which is a public hospital with 6496 beds located in Heilongjiang province between 1 January 2018 and 1 January 2019. The ACS surgeons at the First Affiliated Hospital of Harbin Medical University focus on 3 major specialty areas: trauma, general surgery and orthopedic surgery. The general surgery are subdivided by subspecialty expertise into colorectal, hepatic, breast, thyroid, oncology, gastrointestinal, vascular, pancreatic and endoscopic biliary surgery in our hospital. The orthopedic surgery are subdivided by subspecialty expertise into spine, joint, bone tumour, trauma and hand microsurgery. The bed capacities of the hospital in the general surgery unit, orthopedic surgery unit, ACS unit and intensive care unit are 768, 455, 111 and 78 respectively.

The data were collected from electronic hospital medical records and patient ED charts, including the patients’ demographic characteristics (age, gender and length of hospital stay), need for intensive care unit admission, presenting complain or ED diagnosis and outcome, including discharge and hospitalization rates and the date and in-hospital mortality. The primary analysis was conducted on the entire cohort of patients and included surgical priority (emergent and elective operations). Emergent cases were defined as an operation performed for an emergency medical condition that is expected to result in a severe adverse patient outcome in the absence of surgery (typically performed within 24 h of admission). Elective operations are scheduled in advance, with an outpatient interval between decision to operate and actual operation.

Patients were assigned a diagnostic group based on their principal diagnosis utilizing the Clinical Classifications Software for International Classification of Diseases (ICD), 9th revision. ACS encompasses trauma, surgical critical care, and EGS. While the scope of trauma practice is well established, the last two components remain unclear. There is no unified definition of what constitutes an EGS patient and no easy way to identify them in existing databases, such as E-codes for trauma. So, patients in our study were divided into two groups: Trauma group and Non-Trauma group.

The current study was reviewed and approved by the Research Ethics Committee of the First Affiliated Hospital of Harbin Medical University. All methods were carried out in accordance with guidelines outlined in the Declaration of Helsinki. The requirement for individual patient approval for participation deemed not to be required. Informed consent was waived by the Research Ethics Committee of the First Affiliated Hospital of Harbin Medical University. We collected and analyzed data of patients anonymously.

### Statistical analysis

Data were summarized using descriptive statistics. Mean and standard deviation was presented for continuous variables, and frequencies and percentages for categorical variables. Independent *t*-tests were used to compare means and Pearson’s Chi-square test for groups’ comparison. Binary logistic regression analysis (backward model) was used to identify variables associated with in-hospital mortality. For continuous normally distributed data, the ANOVA test was used. Analysis was carried out using SPSS (Statistical Package for Social Science, IBM SPSS Statistics, Version 23 for Macintosh; IBM Corp., Armonk, NY, USA). An alpha level of 0.05 was considered statistically significant.

## Results

**Figure 2:**
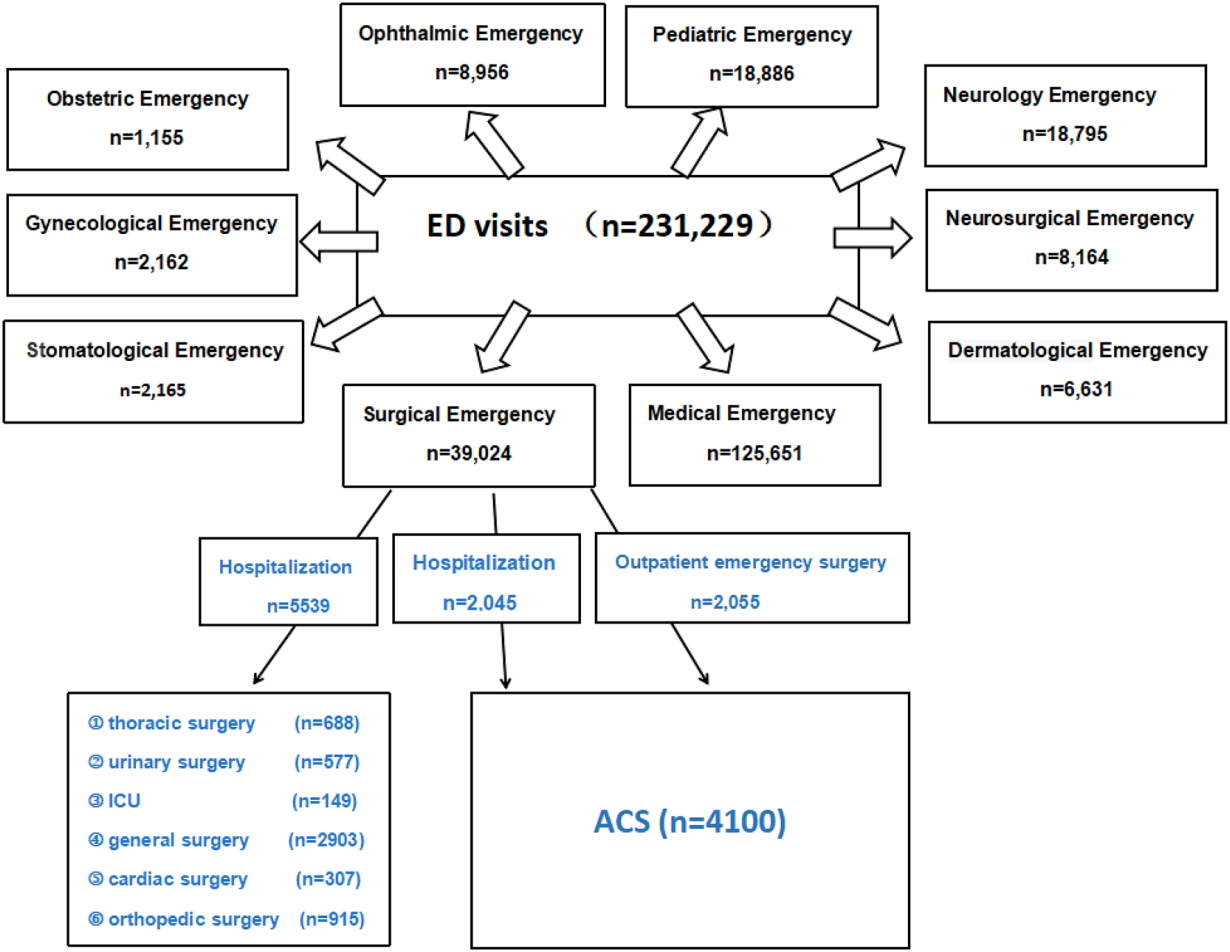
The population and distributing of ED visits at the First Affiliated Hospital of Harbin Medical University between 1 January 2018 and 1 January 2019.

There were ten specialties ED represented in our hospital: surgical emergency, medical emergency, stomatological emergency, gynecological emergency, obstetric emergency, dematological emergency, neurosurgical emergency, neurology emergency, Ophthalmic emergency and pediatric emergency. During the study period there were 231,229 ED visits; 4100 of these patients were admitted for ACS service (Fig. 2). The largest volume of ED visits related to specialty services was in medical emergency, surgical emergency and pediatric emergency, respectively. Among the surgical emergency hospitalized patients, general surgery (52.41%; 2903/5539) and orthopedic surgery (16.52%; 915/5539) were the 2 most frequent sub-specialty and accounted for more than 68% of all sub-specialty.

**Figure 3:**
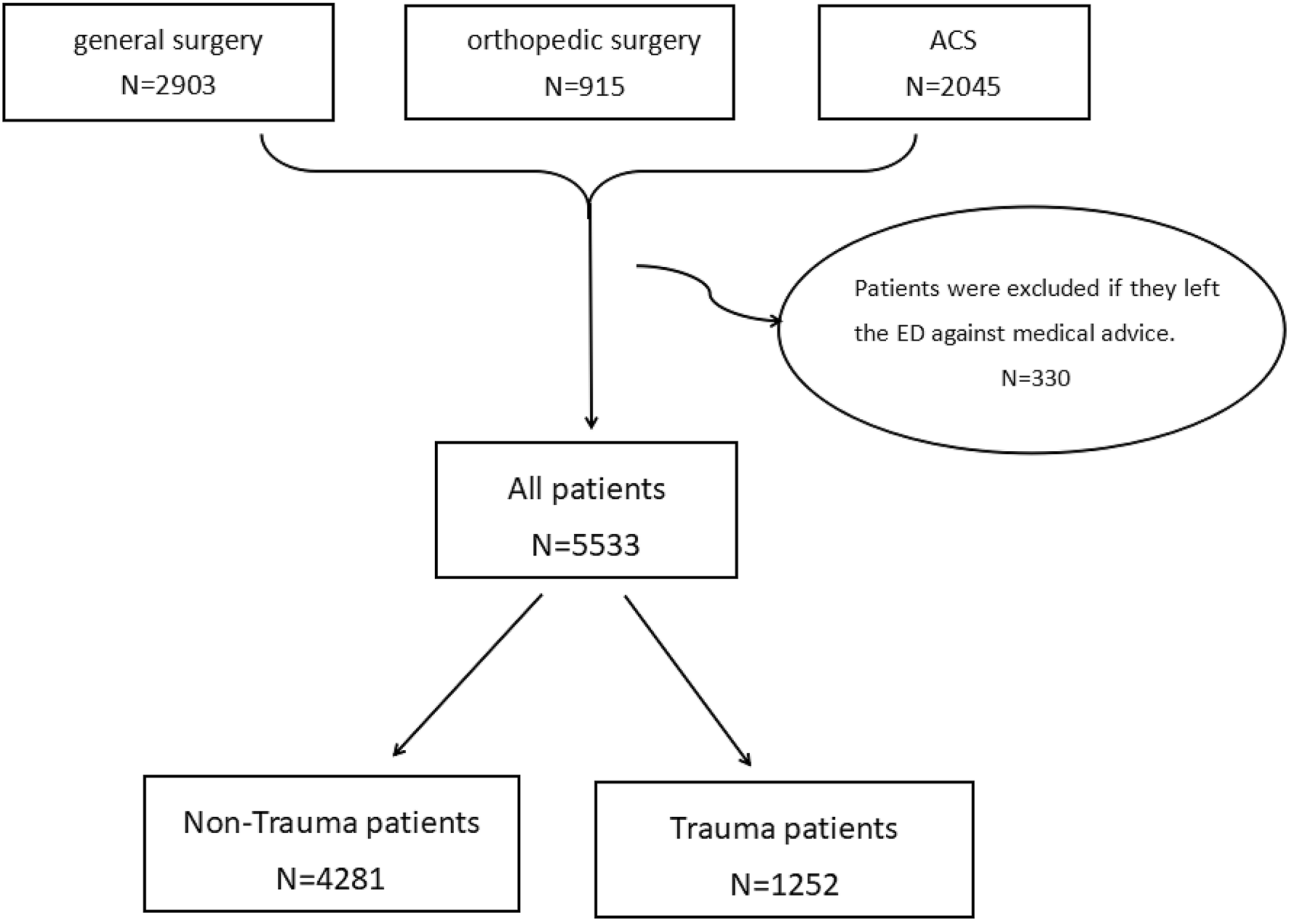
Patients selection process.

**Figure 4:**
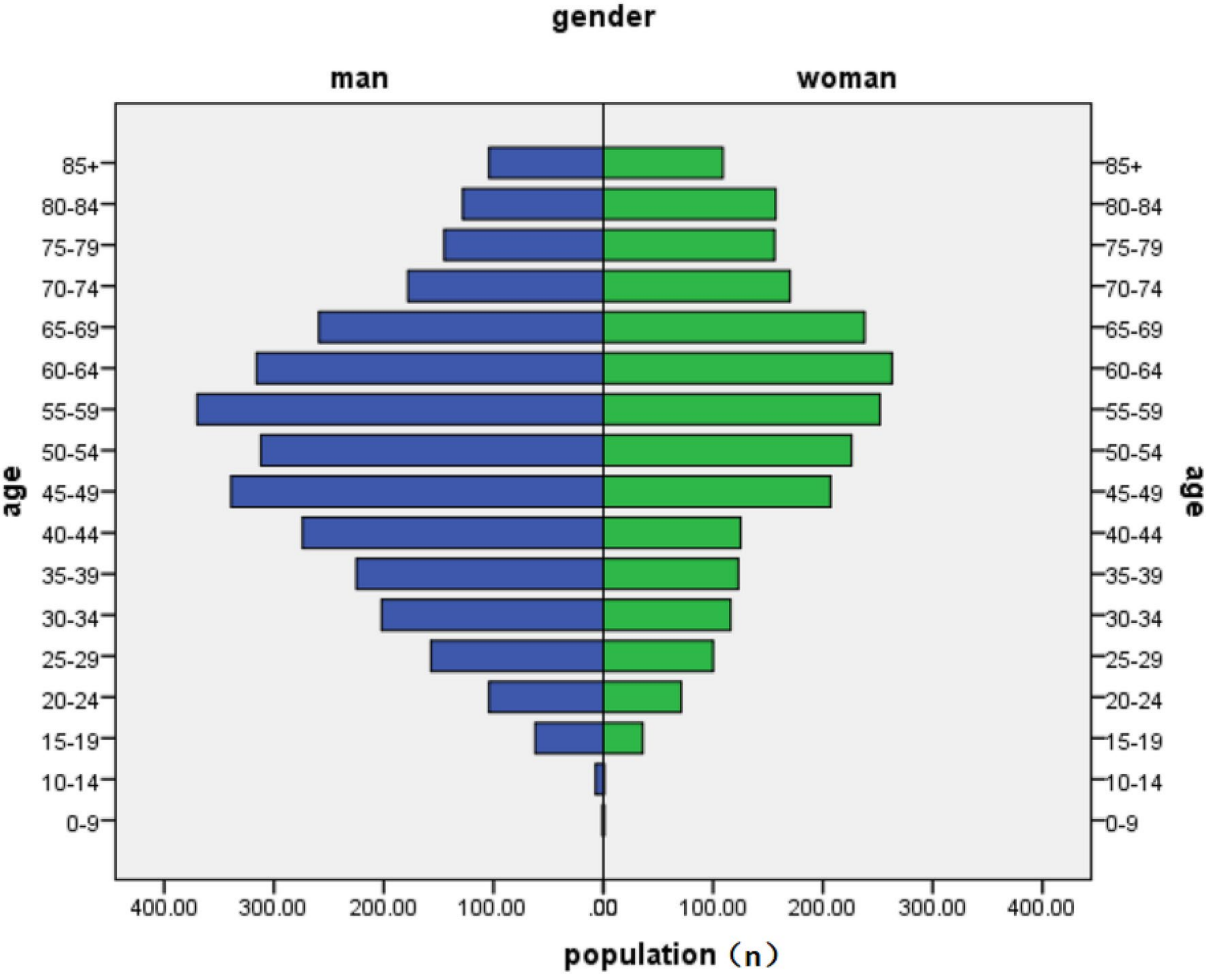
Age pyramid of 5533 patients were selected in our study.

A total of 5533 patients were selected for analysis (Fig. 3). The overall mean age was 52.69 ± 17.42 years. When combined, a total number of 2689 (48.59%) patients had an age range of 0–54 years, 2631 (47.55%) patients were between the ages of 55 and 84 years, and 213 (3.85%) patients were 85 years and older (Fig. 4). There were 1252 patients in Trauma group and 4281 patients in Non-Trauma group. Of the Trauma group, 60.78% were male and 39.22% were female, and for the non-Trauma group these were 56.57% and 43.42% respectively. There was a significantly higher lengths of LOS in Trauma group compared to the non-Trauma group (11.76 ± 13.38 vs. 8.73 ± 7.38, p = 0.000). Two point ninety-nine percent of patients in the non-Trauma group and 2.32% of patients in the Trauma group died in hospital. Nevertheless, the above characteristics were not statistically different between the two groups, even if stratifying patients by age. There were significantly higher proportions of ICU stay in Trauma group compared with the non-Trauma group cohort (8.62% vs. 6.38%, respectively, P = 0.008). Of the Trauma group, 51.6% of patients received operative therapies, and for the non-Trauma group these were 48.05%. There was a trend for a greater proportion of patients in the Trauma group received emergency operative therapies when compared with the non-Trauma group (43.61% vs. 26.77%, respectively, P = 0.000) (Table 1).

**Table 1:** Demographic and operative characteristics of the study population.

Patients characteristics	All patients n = 5533 (%)	Non-trauma patients n = 4281 (%)	Trauma patients n = 1252 (%)	*P*-values
Age (years) ± SD	52.69 ± 17.42	55.73 ± 17.48	49.94 ± 17.52	0.456
Sex (male/female)	3183 (57.53)/2350 (42.47)	2422 (56.57)/1859 (43.42)	761 (60.78)/491 (39.22)	0.008
LOS (days)	10.67 ± 7.27	8.73 ± 7.38	11.76 ± 13.38	0.000
ICU	381 (6.86)	273 (6.38)	108 (8.62)	0.008
**Mortality**	157 (2.84)	128 (2.99)	29 (2.32)	0.245
≦ Aged 54 years	28/2689 (1.04)	17/1922 (0.88)	11/767 (1.43)	0.211
Aged 55–64 years	24/1200 (2.00)	20/960 (2.08)	4/240 (1.67)	0.802
Aged 65–74 years	46/845 (5.44)	40/725 (5.52)	6/120 (5.00)	1.000
Aged 75–84 years	42/586 (7.17)	36/503 (7.16)	6/83 (7.23)	1.000
≧ Aged 85 years	17/213 (7.98)	15/171 (8.77)	2/42 (4.76)	0.535
Surgical operation	2703 (48.85)	2057 (48.05)	646 (51.60)	0.029
**Treatment method**				
Elective operation	1011 (18.27)	911 (21.28)	100 (7.99)	0.000
Emergency operation	1692 (30.58)	1146 (26.77)	546 (43.61)	0.000
Non-operation	2830 (51.14)	2224 (51.95)	606 (48.40)	0.000

Continuous variables are given as mean standard deviation, and categorical variables as number (percentage).

Multivariate analysis identified age ≧ 65 (p = 0.023; odds ratio, OR = 2.66), ACS model (p = 0.000, OR = 0.18), ICU stay (p = 0.000, OR = 118.73) as factors associated with in-hospital mortality (Table 2). Gender, trauma, emergency operation and length of stay (LOS) were not associated with increased mortality in the present study.

**Table 2:** Factors associated with in-hospital mortality (multivariate analysis).

Factors	Multivariate analysis (binary logistic regression)		
	*B*	*P* value	AOR (95% CI)
Male	− 0.462	0.085	0.63 (0.41–0.96)
Age ≥ 65	0.979	0.023	2.66 (1.14–6.19)
ACS	− 1.707	0.000	0.18 (0.07–0.47)
Trauma	0.089	0.751	1.09 (0.63–1.89)
ICU stay	4.777	0.000	118.73 (72.64–194.03)
Emergency operation	− 0.571	0.091	0.56 (0.29–1.09)
LOS	− 0.115	0.622	0.84 (0.42–1.66)

**Figure 5:**
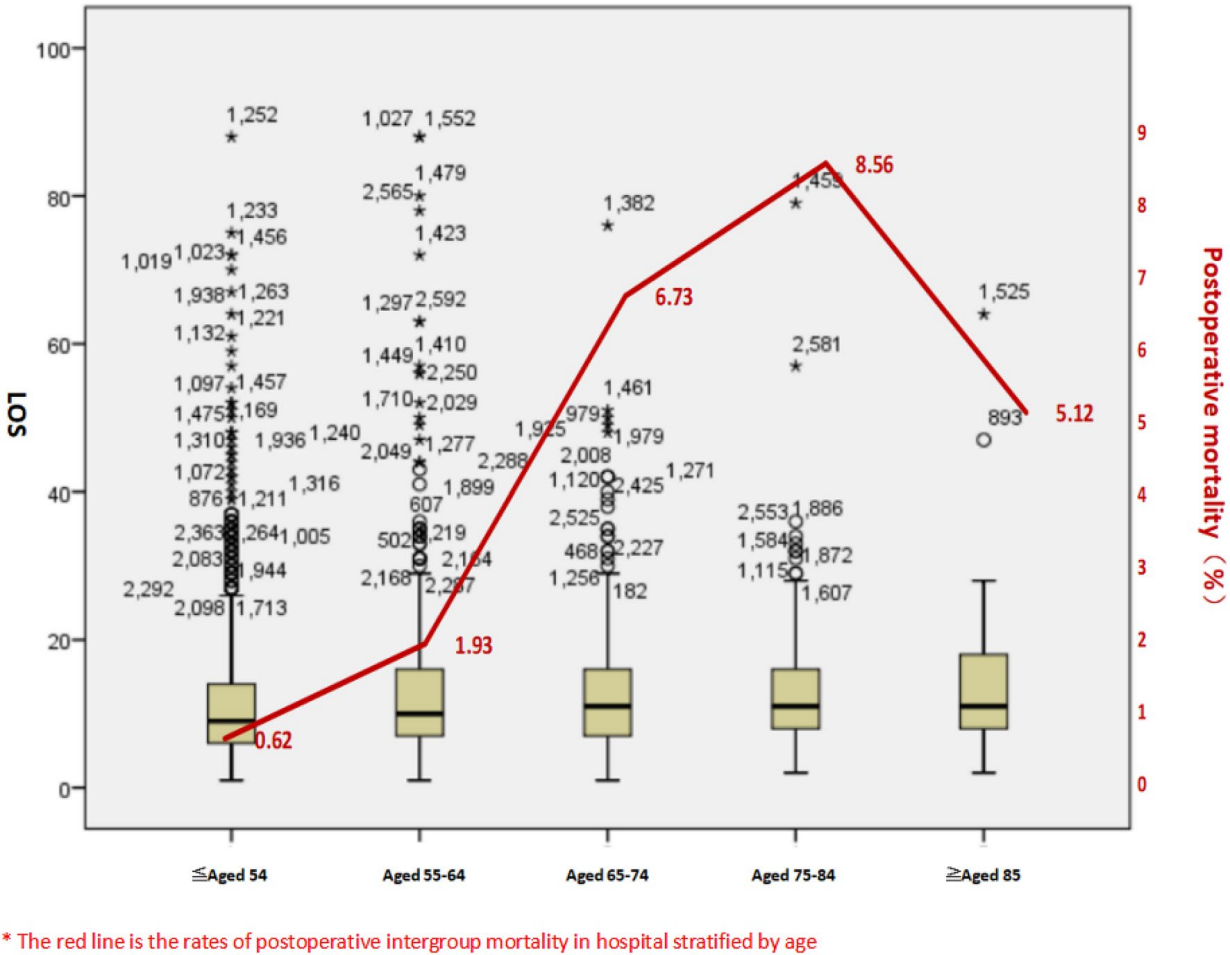
Stem-and-Leaf Plot :overall length of stay of patients stratified by age.

During the study period, we wondered if age factors were contributing to ED length of stay for surgical patients and thereby contributing to overall ED crowding. Therefore, we performed a study to determine the effect of all age ranges patients on overall ED length of stay. Further investigation revealed that most of the assumptions were not statistically significant. However, after stratifying patients by age, we found that there was a increase in LOS between young and elderly postoperative patients (11.67 ± 9.48 vs 13.95 ± 9.11 p < 0.05) (Table 3), meanwhile, the elderly had higher rates of postoperative mortality in hospital (0.62 vs 8.56) (Fig. 5).

**Table 3:** Interrelation analysis of LOS and age among postoperative patients.

	n	LOS (${\overline{\text{x}}} \pm {\text{s}}$)	*F*	*P*-values
≦ Aged 54 years	1291	11.67 ± 9.48	3.622	0.006
Aged 55–64 years	622	12.97 ± 10.64		
Aged 65–74 years	416	12.92 ± 8.64		
Aged 75–84 years	257	13.08 ± 7.97		
≧ Aged 85 years	78	13.95 ± 9.11		

*One-way ANOVA test.

## Discussion

The ACS model was defined as a surgical team dedicated to the evaluation and management of urgent and emergent surgical consultations without having concurrent obligations related to the surgeons’ elective practices. The traditional model of care for emergency surgery patients was an “on-call” system which was defined as having a rotating pool of general surgeons who would intermittently take call while continuing with their regularly scheduled clinical obligations^4^. Previous studies have demonstrated the improvement in injury-related mortality and length of stay in hospital systems that use this ACS model^5^. Furthermore, ACS also facilitates the logistics of caring for the acutely ill patient, maintains trauma surgeons’ operative skills, and allows the elective surgeons to pursue an uninterrupted schedule. Despite these theoretical benefits to the hospital and surgical subspecialist, acute care surgeons often experience relatively some problems and dilemmas involved in exploding ED visits, elderly patients and discipline development^6^. In response to this challenge, a special ACS model was developed at a Third Grade Class A hospital in Harbin, Heilongjiang province, China. The key elements were:

- The ACS surgeons at the First Affiliated Hospital of Harbin Medical University focus on 3 major specialty areas: trauma, general surgery and orthopedic surgery. There are historical and geographic reasons for this arrangement, and hospital data in recent years have shown that the majority of surgical emergency patients belong to general surgery and orthopedic patients. In our study, among the surgical emergency hospitalized patients, general surgery and orthopedic surgery accounted for more than 68% of all sub-specialty.
- The impetus for the ACS target was the desire by speeding recovery to reduce ED overcrowding and hospital access block in the hope of improving both outcomes for patients and health service efficiency.
- ACS surgeons commit to being available for a 24-h emergency duty omnibus diebus quaternis, and to lead a team consisting of a senior surgical resident, several junior residents, and medical students. Another teams were on call the other night. There are total four teams including 20 ACS surgeons in our ACS model.
- In this new model, the sub-specialty staff surgeons were available for a 24-h emergency with ACS surgeons at the same time. It is worth noting that the sub-specialty staff surgeons who on emergency duty do not do elective surgery, but devote themselves to being available in a timely fashion to perform any emergency operation or provide emergency consultation. In our hospital, orthopedics and general surgery involve 14 sub-specialties, each of which is further divided into several medical teams. Each medical team is responsible for one day’s emergency treatment in turn. With the advantage of the preponderance of sub-specialty staff surgeons, this arrangement obviously does not affect the treatment of non-emergency patients and increase job pressure of sub-specialty surgeons.
- ACS surgeons are the dominant force in the treatment of emergency patients, and decide which patients need to be admitted to hospital and the departments in which they are admitted (such as ACS unit, general surgery unit, orthopedic surgery unit, etc.).
- In order to ensure the implementation of the ACS, we create a 24-h emergency outpatient surgery unit at our tertiary referral center. Emergency outpatient surgery differed from ambulatory surgery and emergency day-case surgery, as it implies either did not require hospitalization or a LOS less than 24 h, which could include overnight hospitalization. All emergency outpatient procedures are performed by ACS surgeons.
- Setting targets for LOS of ED patients based on triage acuity, disease characteristics and monthly ED visits. ACS surgeons receive additional funds each month contingent on meeting ACS-specific improvements in achieving targets. This is called pay for performance: the “money”.

### Dilemmas involved in ED visits

ED overcrowding has been defined as a situation in which demand for acute care exceeds the ability of physicians and nurses to provide timely quality care, which threatens patient health and fosters patient dissatisfaction^7^. Many factors can contribute to overcrowding: pre-emergency factors that drive demand for emergency services, patients frequently use ED for non-urgent conditions, size/capacity of the ED and its efficiency of processing, and flow out from the ED to the hospital wards^8^. In many respects, our hospital has noted an increased acuity of ED visits presenting to us. There were 231,229 ED visits; 7584 surgical emergency patients of these were hospitalization, corresponding to 21 surgical emergency patients were hospitalization every day; 4100 of these patients were admitted for ACS service between 01 January 2018 and 1 January 2019. Faced with surge in ED visits, we think that ED crowding is most commonly due to the boarding of inpatients lacking beds for extended periods–reducing or eliminating this should greatly improve ED efficiency. It’s remarkable that saving 1–2 days per treated patient, hospital bed utilization will be reduced by 15%. So, we made some adjustments on the ACS model (see the key elements), and we think this will be more conductive to reducing the LOS and increasing the ACS’ bed utilization rate.

Non-operative management (NOM) has been advocated for years in several areas of blunt injury, such as blunt chest or abdominal trauma, and has achieved a success rate of 90%^9^. According to recent studies, NOM could even be considered for patients with cardiovascular injury or fractures involving the thoracic or lumbar spine^10,11^. This success has accomplished as a result of advances in ACS, high-quality imaging, interventional radiology techniques and relies on a well-established ICU and the timely backup of the surgical team. Some researchers also reveal that selective NOM reduces the LOS for penetrating abdominal injury patients as well as the rate of non-therapeutic laparotomies and concomitant complications^12^. In our study, there are almost 50% non-surgical treatments in both groups (trauma and non-trauma, 51.6% vs 48.05%). From our experience, patient selection may have an impact not only on the rate of failure of NOM, but the mortality rate associated with it. Nowadays, the presence of hemodynamic instability is considered the only absolute contraindication for NOM^13^. Ongoing research is needed to evaluate whether to treat the patient operatively or with selective NOM, potentially leading to reduced LOS.

Moreover, timing of surgical intervention is also critical for outcomes of patients diagnosed with surgical emergencies. Facing the challenge of multiple patients requiring emergency surgery, ACS surgeons as the triage officer in our hospital should triage patients according to their disease process and physiological state. We propose that the use of a color-triage system which recommended by The World Society for Emergency Surgery (WSES) to triage emergency surgery cases may help to reduce information loss, time spent on conferring with other caregivers regarding scheduling of emergency operations and improve quality of care^14^.

### Dilemmas involved in elderly patients

As is known to all, The World Health Organization (WHO) defines an elderly person as one who has a chronological age of 65 years or more. Life expectancy and the proportion of the elderly population are increasing worldwide. The population in China is also aging rapidly. According to the “2018 China Statistical Yearbook Compiled by National Bureau of Statistics of China (http://www.stats.gov.cn/),” people aged 65 and over accounted for 11.4% (158.31 million) of the total population in 2017 in China; by 2030, the population is predicted to be 1.46 billion, and 16% percent of Chinese citizens will be aged 65 and over^15^. In our study, more than half of patients were over 55 (52.41%), and 213 patients were 85 years and older (Fig. 2). Recent research has highlighted the importance of biological not just chronological age. Functional decline and chronic health conditions can appear earlier than 65 years, particularly among individuals who experience health disparities^16^. The increasing rate of serious injury among older adults represents a formidable challenge for geriatric care as elders fare worse in terms of risk of disability, hospital length of stay, and risk of mortality in comparison with younger individuals with similar injuries. For example, Sieling et al. found that among the oldest old undergoing surgery for trauma, 8% of the patients experienced at least one medical complication, whereas 5% of the patients experienced multiple complications, including heart arrhythmias requiring stabilization, respiratory failure requiring ventilation, gastrointestinal tract bleeding, and urinary tract infections^17^. The issue of mortality is an important one in emergency surgery, especially in elderly patients. In our study, Multivariate analysis identified age ≧ 65 (p = 0.023; odds ratio, OR = 2.66) is the factor associated with in-hospital mortality. The postoperative mortality increased with age is more pronounced, when stratifying patients by age. What is noteworthy is that there was also a increase in LOS between young and elderly postoperative patients (11.67 ± 9.48 vs 13.95 ± 9.11 p < 0.05). In our ACS model, we settle the problem of geriatric emergency patients by transfering some complicated diseases or the oldest old patients to the highly specialized surgical units.

In our hospital, the general surgery are subdivided by subspecialty expertise into colorectal, hepatic, breast, thyroid, oncology, gastrointestinal, vascular, pancreatic and endoscopic biliary surgery. The orthopedic surgery are subdivided by subspecialty expertise into spine, joint, bone tumour, trauma and hand microsurgery. As is known to all, surgeons are more often tending to “organ-specific” surgeon practices (“breast surgeon”; “pancreatic surgeon”; “colorectal surgeon”; “gastrointestinal surgeon”). Highly focused specialization in all aspects of medicine is accelerating as the complexities of care increase and the sophisticated demands of patients show no sign of abating. This has led a shift in focus from general surgery with broad spectrum of diseases and surgical techniques. Meanwhile medical specialization is increasingly being managed by minimal-invasive techniques^18^. Overall, the general surgery workforce has followed a trend of increased specialization. The era of the “omnipotent general surgeon” is to an end and in order to improve quality in specific areas many institutions dedicate most of their economic resources to highly specialized surgical units. However, in a emergency situation, best specialist for the patients must be the ACS, given the idiosyncrasy and peculiarity of its diagnostic and therapeutic management. ACS surgeons should comprehensive evaluate patients according to their disease process and physiological state. At the same time, by transfering some complicated diseases or the oldest old patients to the highly specialized surgical units, this can also increase the ACS’bed utilization rate.

### Dilemmas involved in discipline development

Though not currently well-described in the literature, many models across the world essentially added EGS services to existing trauma services, creating a hybrid trauma-EGS services. In China, ACS hospitals are concentrated in large academic referral centers. Although past studies have demonstrated improvements in efficacy at such institutions with ACS implementation, most of them appear to the hybrid model. In fact, in most of the world, general surgeons associated to other clinicians in a dedicated team to ACS patients, practice ACS by necessity and not based on specific training in the specialty. Focus on the facts that several models must be created based on patients’ needs and fitted to the local/regional public health requirements. Compared with this hybrid model, the standard ACS model requires surgeons to acquire more skills, which often involve multiple specialties. This has resulted in heavy pressure on ACS surgeons and no formal ACS specialists in most countries. The danger ACS surgeons (and thereby trauma centers and systems) face is that they may attract few new recruits unless we provide a clear advantage and meaningful purpose to this career choice. So we first came up with this concept of Fast Track Acute Care Surgery (FTACS) through the implementation of this special model.

Enhanced Recovery after Surgery (ERAS) programmes, also referred to as “fast track”(FT) perioperative care, are evidenced-based protocols designed to standardize and optimize perioperative care in order to reduce surgical trauma, perioperative physiological stress and organ dysfunction (metabolic, endocrine and inflammatory response as well as reduce protein catabolism) related to elective procedures^19^. Nowadays, a systematic review focusing on safety of ERAS for geriatric emergency patients was conducted by Paduraru et al.^20^. Their study demonstrated that ERAS can be safely applied to emergency patients with a reduction in postoperative complications, hospitalization and readmission rates. Patient compliance was also addressed in a number of the studies reviewed and ranged from 50 to 85%^21,22^. Emergency surgery patients achieved an average level of compliance in comparison to elective surgical patients, again demonstrating that ERAS in emergency surgery is feasible. In our study, the impetus for the FTACS target was the desire by speeding recovery to reduce ED overcrowding and increase the ACS’ bed utilization rate. To some extent, ACS surgeons have a new job goal, that is, focus on clinical research that can speed up the recovery of surgical emergency hospitalized patients. Certainly, one of the most challenging aspects of FTACS improvement is the selection of appropriate patient. In our new model, the initial vision is to triage some emergency patients and keep those patients suitable for rapid rehabilitation treatment in ACS. However, several challenges and opportunities associated with the implementation of this model have yet to be evaluated and overcome before a wider implementation. More research is still needed in relation to identifying which elements of ERAS have greater impact on emergency surgery patients and whether independent impact plays a more significant role.

Furthermore, the protocols of ERAS have been best applied in conjunction with minimally invasive and laparoscopic procedures. Over the second part of twentieth century instead, laparoscopy has increasingly gained success in elective surgery, and from the beginning of the 21th century, it has been introduced also in emergency surgery. Supported by recent scientific literature, laparoscopy has changed the approach to acute cholecystitis and is now recognized as the standard of care in the management of acute appendicitis^23^. In addition, many other emergency conditions can be effectively managed laparoscopically, including trauma in select hemodynamically-stable patients^24^. Certainly, in order to achieve good clinical outcome, it requires a highly skilled laparoscopic ACS surgeon. Training on related projects is under way in our hospital. It is worth noting that despite being advocates of the use of minimally invasive surgery (MIS) in emergency surgery and even in selected cases of trauma surgery, some researchers feel safer performing open surgery as much as possible, both as health care operators and for the patients, especially during the COVID pandemic^25^.

This study has a few limitations. First, it considered only one specialty care unit and ED at the same hospital, so similar studies in other settings might be conducted before forming broad conclusions. Other limitations include that this was a single center study at a large academic medical center in a metropolitan area with a large number of affiliated specialty services. The percentage of patients is likely higher than it would be in a community hospital. Finally, it is generally believed that both preoperative medical conditions and postoperative complications are important determinants for postoperative short- and long-term mortality of emergency patients. In our statistical analysis, we did not provide the details evidence indicates that intraoperative massive blood loss, blood transfusion, adverse cardiovascular events and total fluid volume are associated significantly with increased risks of postoperative mortality after emergency surgery. Our study does also not evaluate for differences in rates of operative, conservative, or interventional management, which could have large impacts on lengths of stay and outcomes.

## Conclusion

ACS is an evolving concept which has not yet found its proper position in the surgical panorama. The data in this article are encouraging for ACS surgeons and should lead other groups to develop their own ACS models and to prospectively collect and pool their data to validate this series. In conclusion, we thought this reform is a bold attempt, even if it tries to break the existing balance between emergency physicians and specialists. The development of new clinical techniques is a slow process, but the benefits of a change in the pattern of diagnosis and treatment can quickly become apparent. Continuous and high-quality surveillance data across China are needed to estimate the acute care surgery model which used to deal with ED overcrowding.

## Data Availability

All data are fully available without restriction.

## Abbreviations

ACS: Acute care surgery
LMICs: Low-income and middle-income countries
EGS: Emergency general surgery
AAST: The American association for the surgery of trauma
ED: Emergency department
ICD: International classification of diseases
LOS: Length of stay
FTACS: Fast track acute care surgery
ERAS: Enhanced recovery after surgery
